# Cause-effect relations between 55 kD soluble TNF receptor concentrations and specific and unspecific symptoms in a patient with mild SLE disease activity: an exploratory time series analysis study

**DOI:** 10.1186/s13104-015-1398-z

**Published:** 2015-09-21

**Authors:** Christian Schubert, Julia Haberkorn, Francisco M. Ocaña-Peinado, Paul König, Norbert Sepp, Mirjam Schnapka-Köpf, Dietmar Fuchs

**Affiliations:** Clinical Department of Medical Psychology, Innsbruck Medical University, Schöpfstraße 23a, 6020 Innsbruck, Austria; Department of Statistics and Operations Research, University of Granada, Granada, Spain; Clinical Department of Internal Medicine, Innsbruck Medical University, Innsbruck, Austria; Clinical Department of Dermatology, Innsbruck Medical University, Innsbruck, Austria; Central Institute of Medical and Chemical Laboratory Diagnostics, University Clinics, Innsbruck, Austria; Division of Biological Chemistry, Biocenter, Innsbruck Medical University, Innsbruck, Austria

**Keywords:** sTNF-R55, Lupus, Proteinuria, Oral ulcer, Facial rash, Time series analysis, ARIMA modeling, Cross-correlation, Single case study

## Abstract

**Background:**

This integrative single-case study investigated the 12 h-to-12 h cause-effect relations between 55 kD soluble tumor necrosis factor receptor type 1 (sTNF-R55) and specific and unspecific symptoms in a 52-year-old Caucasian woman with mild systemic lupus erythematosus (SLE) disease activity.

**Methods:**

The patient collected her entire urine for 56 days in 12 h-intervals to determine sTNF-R55/creatinine and protein/creatinine levels (ELISA, HPLC). Additionally, twice a day, she took notes on oral ulceration and facial rash; answered questionnaires (VAS) on fatigue, weakness, and joint pain; and measured body temperature orally. Time series analysis consisted of ARIMA modeling and cross-correlational analyses (significance level = p < 0.05).

**Results:**

Time series analysis revealed both a circadian and a circasemiseptan rhythm in the urinary sTNF-R55 data. Moreover, several significant lagged correlations between urinary sTNF-R55 concentrations and SLE symptoms in both directions of effect were identified. Specifically, increased urinary sTNF-R55 concentrations preceded decreased urinary protein levels by 36–48 h (r = −0.213) and, in the opposite direction of effect, increased protein levels preceded increased sTNF-R55 concentrations by 24–36 h (r = +0.202). In addition, increased urinary sTNF-R55 levels preceded increased oral ulcers by 36–48 h (r = +0.277) and, conversely, increased oral ulceration preceded decreased sTNF-R55 levels by 36–48 h (r = −0.313). Moreover, increased urinary sTNF-R55 levels preceded decreased facial rash by 36–48 h (r = −0.223) and followed increased body temperature after 36–48 h (r = +0.209). Weakness, fatigue and joint pain were not significantly correlated with urinary sTNF-R55 levels.

**Conclusions:**

This study gathered first evidence of real-life, long-term feedback loops between cytokines and SLE symptoms in mild SLE disease activity. Such insights into the potential role of sTNF-R55 in SLE would not have been possible had we applied a pre-post design group study. These findings require replication before firm conclusions can be drawn.

## Background

One of the pro-inflammatory cytokines thought to be related to the pathogenesis of systemic lupus erythematosus (SLE) and other inflammatory diseases is tumor necrosis factor-α (TNFα) [1]. However, evidence of an association between TNFα and SLE disease activity is inconsistent. Some studies have shown that serum TNFα levels are elevated in SLE patients and correlate with disease activity. Other studies, however, have shown that elevated TNFα plasma levels do not correlate with SLE disease activity or that TNFα levels are actually higher in patients with inactive disease, thus suggesting a protective role of TNFα in SLE [2].

One possible explanation for this heterogeneity would be that TNFα acts via two distinct soluble receptors, 55 kD (sTNF-R55) and 75 kD (sTNF-R75) [3], rendering a pure TNFα effect difficult to detect. Indeed, studies have shown that sTNF-R55 is associated with clinical and subclinical SLE disease activity [4, 5] and that sTNF-R75 may even be an antagonist of TNFα [6]. However, experimental work suggests that both soluble TNF receptor subtypes can act antagonistically to TNFα bioactivity [7], which adds to the complexity of the topic. Another reason for the heterogeneous findings concerning a link between TNFα and SLE disease activity could be that clinical and experimental approaches to this topic have thus far been conventional group studies, which do not consider the dynamic characteristics of cytokine–disease interactions [8]. For example, a lack of correlation between TNFα and SLE symptom in a pre–post design group study does not automatically mean that TNFα is not connected with disease activity. Instead, the effect of TNFα on symptom manifestation may be temporally delayed, appearing later than expected. Furthermore, TNFα might interfere with SLE symptoms, and conversely, SLE symptoms might influence TNFα levels.

This integrative single-case study sought to shed light on the functional role of TNFα on SLE symptom manifestation in everyday life. It used time series analysis to elucidate the 12 h-to-12 h cause-effect relationships between urinary sTNF-R55 concentrations and manifestations of several American College of Rheumatology (ACR)-related symptoms specific and unspecific to SLE, i.e. oral ulcers, facial rash, arthralgia, fatigue, weakness, body temperature, and urinary protein levels [9]. For this purpose, SLE disease activity had to be stable, with only minor symptoms that did not require anti-inflammatory medication. Such medication could have disturbed the naturally occurring interdependencies between the variables under study. In order to have enough power for statistical time series analysis, to consider the chronobiological^1^ characteristics of variables (e.g. circadian rhythm), and to interfere, e.g. through frequent blood draws, as little as possible with the patient’s normal routine, sTNF-R55 levels were determined in over 100 consecutive 12-h urine samples as a non-invasive way to monitor cytokine activity serially. The complete 12 h-to-12 h collection of urine without gaps between measurements is an important advantage over other multiple assessment approaches, such as the Experience Sampling Method (ESM) and the Ecological Momentary Assessment (EMA) [10].

## Methods

### Study design

This single-case study is part of a larger project investigating the effects of emotionally meaningful everyday incidents on stress system parameters in SLE patients [11–13]. At study start, the patient, who had been diagnosed with SLE 8 years earlier, was examined thoroughly using the Systemic Lupus Activity Measure (SLAM) to ensure that her SLE was stable and that no therapy was required. Then, during the following 56 days, the patient collected her entire urine in 12-h intervals (from approx. 8 am to 8 pm and from approx. 8 pm to 8 am; total: 112 12-h units). She also filled out questionnaires twice a day, at approx. 8 am and 8 pm. Weekly, the patient brought the frozen (−20 °C) urine samples to the laboratory, where they were stored at −70 °C until measurement. Moreover, each week, an interview was conducted with her to identify the previous week’s incidents, and she was examined physically to check general health and to detect any signs of SLE disease activity (SLAM).

The patient gave informed consent to study participation and publication of data. The Ethics Committee of the Medical Faculty of the University of Innsbruck approved the design.

### Patient history

At study start in 1997, the patient was a 52-year-old Caucasian woman and non-smoker. In 1989, 8 years prior to the study start, the patient had been admitted to the Department of Internal Medicine, University Hospital Innsbruck and diagnosed with SLE according to the following ARC criteria: kidney involvement with microscopic hematuria (biopsy-confirmed chronic mesangial proliferative glomerulonephritis, WHO classification IIIa [14]), leukopenia, elevated antinuclear antibodies (ANA, 1:2560), mucosal ulcers, and facial skin rash. In addition, the patient had presented with hypocomplementemia, urticarial vasculitis, and arthralgias. Analyses of antinuclear anti-double-stranded DNA antibodies (ds DNA) were negative. Pharmacologic treatment lasted 3 years and consisted primarily of steroids (4–20 mg) in combination with non-steroidal anti-inflammatory medication (paracetamol). Although her disease fit WHO classification IIIa with proteinuria and microhematuria, the patient refused additional immune suppressive therapy (e.g. azathioprine, mycophenolate, cyclophosphamide). The patient did not tolerate antimalarials. Despite the lack of additional immune suppressive regimen, her laboratory values improved (no proteinuria, no pathological urine sediment). Occasionally, however, the following symptoms were seen during regular check-ups between first diagnosis in 1989 and study start in 1997: short-term urticarial vasculitis and facial skin rash, rarely occurring mouth ulcerations, mild daily arthralgia, fatigue, and weakness. These symptoms did not require long-term steroid drug therapy and were treated by the patient symptomatically (e.g. using pain relief medication). Her condition was thus stable, with no progression of SLE disease. At study start, the patient presented with elevated ANA (1:160, ds DNA negative, SS-Ro-antibody positive) with the above-mentioned mild clinical symptoms not requiring steroid treatment.

### Measurement of fatigue, weakness, small joint pain, oral ulceration, facial rash, and body temperature

Twice each day, using 100 mm visual analogue scales (VAS), the patient rated the intensity of her fatigue, weakness, and small joint pain. Also, every 12 h, she recorded any mucosal and cutaneous manifestations (i.e. oral ulcers, facial rash) and rated their intensity via VAS. These VAS are part of the DIARI, a paper-and-pencil questionnaire that was also used by the patient to note in 12-h intervals any sporadic drug use/medication and potential signs of a cold, flu, etc. [11–13]. Additionally, she measured her body temperature orally for approx. 120 s using a commercially available mercury thermometer with a scale interval of 0.1 °C (model no. 1711, Scheiber GmbH, Kreuzwertheim, Germany). From these 12 h-to-12 h measurements of fatigue, weakness, small joint pain, oral ulceration, facial rash, and body temperature, various time series were constructed.

### Measurement of urinary sTNF-R55 and creatinine levels

Urine samples were stored at −70 °C until analysis. Within 3 months following urine collection, the 112 consecutive urinary sTNF-R55 levels were measured in a single run using ELISA (Quantikine™, R&D Systems, Minneapolis, MN, USA). The kit instructions state that neither recombinant human TNF-α nor recombinant mouse TNF-α show any significant cross-reactivity with sTNF-R55. For time series analysis, TNF-R55 concentrations were expressed in micromolar per molar (μmol/mol) creatinine. Urinary creatinine levels were measured via High Pressure Liquid Chromatography (HPLC) (Model LC 550; Varian Associates, Palo Alto, CA, USA), as previously described [15]. In this study, sTNF-R55 concentrations were measured three times, using a new aliquot for each of the three independent determinations. Results were averaged to reduce variation of analytical performance.

### Measurement of urinary protein

The amount of protein in each 12-h urine sample was measured in milligrams per deciliter (mg %) using the benzethonium chloride method [16] at 505 nm with a Hitachi 911 analyzer (Roche). The time series of urinary protein concentrations was expressed in μmol per mol creatinine.

### Time series analysis

A detailed description of the statistical analyses used in this study is given elsewhere [8]. In short, cross-correlational analyses between sTNF-R55 levels and the ACR-related criteria under study were performed at lag0 and at higher lags up to ±7 using SPSS-Trends™ 21.0 [17]. We controlled for spurious cross-correlations due to trends (e.g. circadian rhythm) and serial dependencies (e.g. autoregression) by cross-correlating residuals’ series after autoregressive integrated moving average (ARIMA) modeling of the time series [18]. This is a rather conservative approach that lowers effect sizes and makes it harder to achieve significant findings compared to cross-correlational analysis without data modeling.

Autoregressive integrated moving average modeling was based on the following procedural specifics: when the mean of a series needed to be stabilized, a deterministic trend was either removed from the series, or the series was differenced. When the variance of a series needed to be stabilized, the series was transformed (e.g. log, square root). Transformation of time series was also used to improve model specification. Moreover, a time series that did not need to be modeled and was proved to be not normally distributed was transformed before cross-correlation. In case of missing values within a time-series, linear interpolation was performed. The level α of statistical significance was *p* < 0.05. As our single-case studies, at this stage, have more exploratory than confirmatory character, we did not perform p-value adjustments for multiple testing in order to preserve flexibility in design and analysis [8].

## Results

**Fig. 1:**
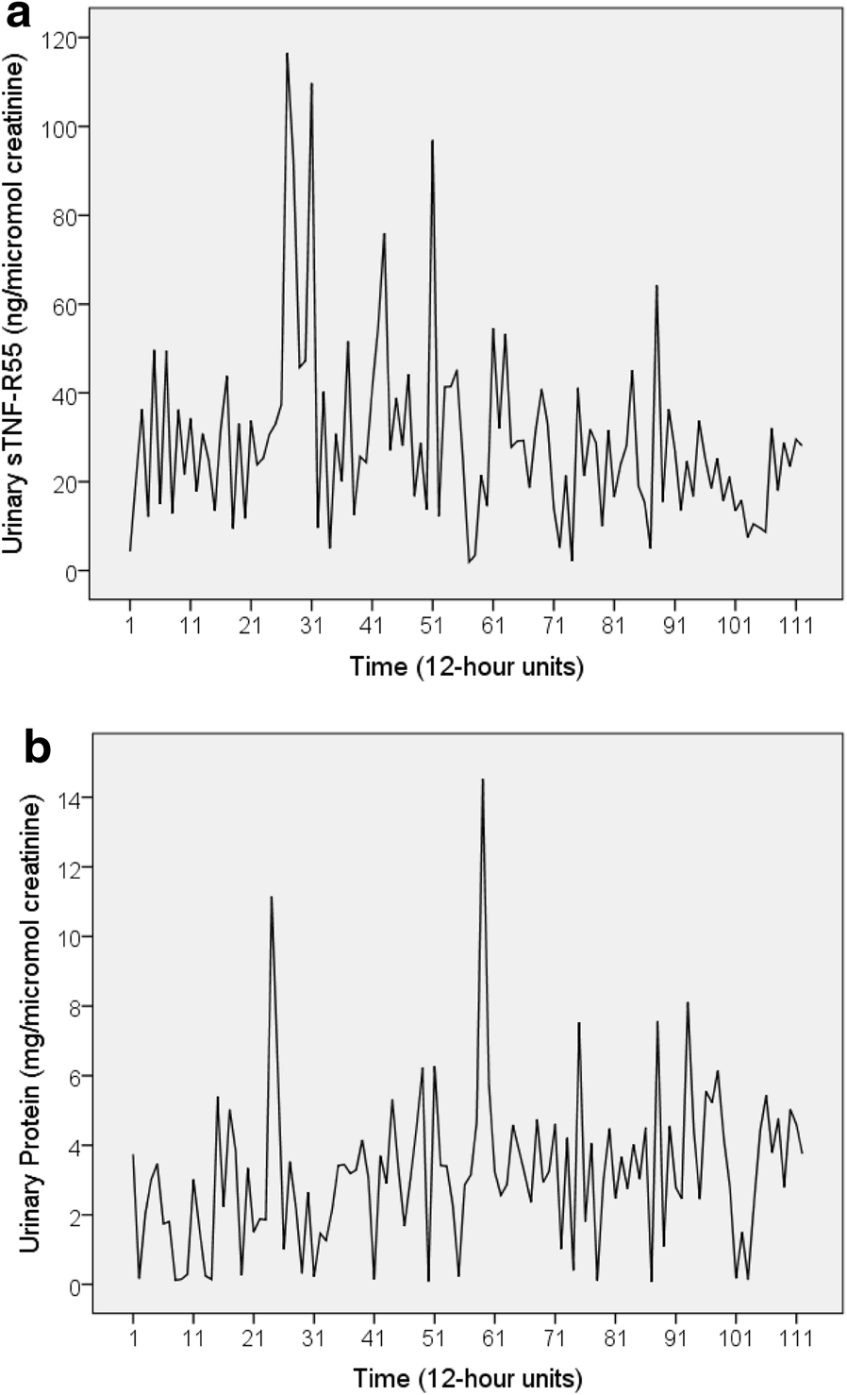
Time series of urinary sTNF-R55 levels and urinary protein levels of the SLE patient under study. **a** Time series of urinary sTNF-R55 (ng per μmol creatinine), **b** time series of urinary protein (mg per μmol creatinine). Both time series cover a period of 56 days during which the subject collected her entire urine in 12-h intervals. Each of the 112 12-h units represents either a daytime period from 8 am to 8 pm (*uneven numbers*) or a nighttime period from 8 pm to 8 am (*even numbers*)

Table 1 shows the mean, standard deviation, and range of all variables under study. The weakness time series had one missing value (at 12-h unit 52); all other times series were complete, i.e. contained 112 measurements. The three independently determined urinary sTNF-R55 time series were strongly correlated (r = 0.821–0.971), suggesting high analytical precision of measurement. The ARIMA model of the urinary sTNF-R55 time series was AR(2) SMA(1), s = 14, ln. Table 2 shows all findings from univariate time series analyses. The patient indicated having mild facial rash on 26 of 112 12-h units (23 %) (maximum intensity: 22 %; maximum duration: 60 h). On 6 of 112 12-h units (5 %), she had minor manifestations of oral ulcers (maximal intensity: 19.9 %; maximal duration: 24 h). Figure 1a, b shows the time series of urinary sTNF-R55 and urinary protein concentrations (both in μmol per mol creatinine). Halfway through the study period (between 12-h units 45–54), the patient suffered from acute paranasal sinusitis [11–13]. Comparison of grouped time series data using the Mann–Whitney U test revealed that only weakness levels differed significantly between the periods within and outside the inflammatory event. The weakness time series remained heteroskedastic even after log transformation (data not shown). Neither the weekly clinical check-ups nor the 12 h-to-12 h notes made by the patient revealed any signs of infection and/or increased SLE activity during the study period.

**Table 1:** Descriptive statistics of urinary sTNF-R55 concentrations and SLE-specific and SLE-unspecific symptoms (N = 112 consecutive measurements)

Parameter	Mean ± SD	Range
Urinary sTNF-R55 (ng/μmol creatinine)	29.2 ± 20.1	1.94–117
Urinary sTNF-R55 (pg/ml)	277 ± 183	52.0–861
Urinary sTNF-R55 (ng/h)	16.7 ± 11.5	0.94–81.1
Urinary protein (mg/μmol creatinine)	3.20 ± 2.25	0.08–14.5
Urinary protein (mg/dl)	3.48 ± 2.58	0.10–10.0
Urinary protein (mg/h)	1.76 ± 1.09	0.07–6.38
Oral ulceration (%)	0.59 ± 2.79	0.00–19.9
Facial rash (%)	2.67 ± 5.60	0.00–22.0
Body temperature (°C)	36.7 ± 0.25	36.1–37.4
Weakness (%)	7.77 ± 5.86	0.00–38.0
Fatigue (%)	35.2 ± 17.2	6.00–77.0
Joint pain (%)	20.2 ± 11.5	5.00–54.0

*SD* standard deviation, *sTNF-R55* soluble tumor necrosis factor 55 kD receptor

**Table 2:** Summary of findings including ARIMA models and cross-correlation results between sTNF-R55 concentrations and SLE-specific and SLE-unspecific symptoms

	Urinary sTNF-R55, AR(2) SMA(1), s = 14, ln	
Urinary protein, (0,0,0), cube root	−lag2: r = +0.202; *p* = 0.033	+lag3: r = −0.213; *p* = 0.024
Oral ulceration, binary-coded	−lag3: r = −0.313; *p* = 0.001	+lag3: r = +0.277; *p* = 0.003
Facial rash, binary-coded	n.s.	+lag3: r = −0.223; *p* = 0.018
Body temperature, deterministic season, s = 2, ln	−lag3: r = +0.209; *p* = 0.027	n.s.
Weakness, MA(4), ln	n.s.	n.s.
Fatigue, deterministic trend	n.s.	n.s.
Joint pain, AR(1), deterministic trend	n.s.	n.s.

+Lag means that sTNF-R55 levels precede SLE symptom, and –lag means that SLE symptom precedes sTNF-R55 levels

*sTNF-R55* soluble tumor necrosis factor 55 kD receptor, *AR* autoregressive, *MA* moving average, *SMA* seasonal moving average, *s* seasonality, *n.s.* not significant

**Fig. 2:**
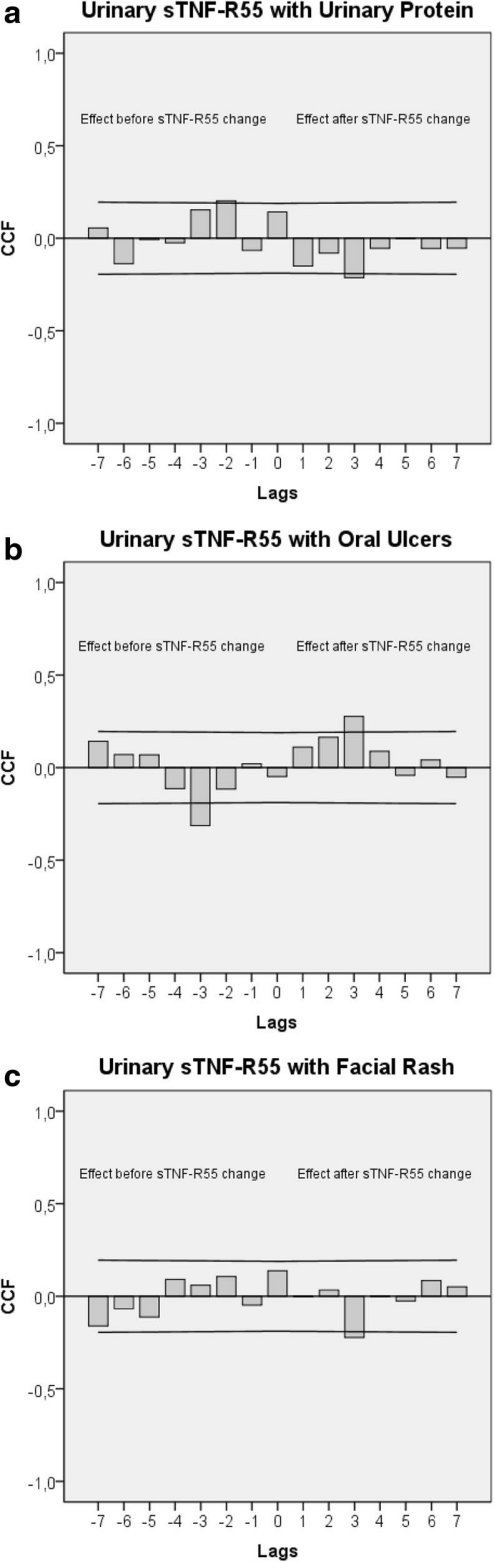
Cross-correlational functions (CCF) between urinary sTNF-R55 levels and SLE-related symptoms. **a** Urinary sTNF-R55 levels with urinary protein, **b** urinary sTNF-R55 levels with oral ulcers (binary-coded), **c** urinary sTNF-R55 levels with facial rash (binary-coded). Each lag represents a time interval of 12 h. *Plots* show cross-correlation coefficients (*bars*) as well as upper and lower limits of the 95 % confidence intervals. Significance level is *p* < 0.05. A positive lag significance means that urinary sTNF-R55 levels precede SLE-related symptoms (*effect after sTNF*-*R55 change*), whereas a negative lag significance means that SLE-related symptoms precede urinary sTNF-R55 levels (*effect before sTNF*-*R55 change*). In **a** and **b** there is a clear change in the sign of the cross-correlation function between positive and negative lags, indicating negative feedback loops

The cross-correlogram in Fig. 2a shows that increased urinary sTNF-R55 concentrations preceded decreased urinary protein levels by 36–48 h (lag3: r = −0.213; *p* = 0.024) and that, in the opposite direction of effect, increased urinary protein levels preceded increased urinary sTNF-R55 concentrations by 24–36 h (lag2: r = +0.202; *p* = 0.033). Figure 2b shows that increases in urinary sTNF-R55 levels preceded increases in oral ulcers (binary-coded) by 36–48 h (lag3: r = +0.277; *p* = 0.003) and that, in the opposite direction of effect, increases in oral ulceration (binary-coded) preceded decreases in urinary sTNF-R55 levels by 36–48 h (lag3: r = −0.313; *p* = 0.001). Figure 2c shows that increased urinary sTNF-R55 concentrations preceded decreases in facial rash (binary-coded) by 36–48 h (lag3: r = −0.223; *p* = 0.018). Moreover, increases in urinary sTNF-R55 levels followed increased body temperature after 36–48 h (lag3: r = +0.209; *p* = 0.027) (data not shown). Weakness, fatigue and joint pain were not significantly correlated with urinary sTNF-R55 concentrations, in either direction of effect. Table 2 summarizes the bivariate findings of this study.

## Discussion

This integrative single-case study on a 52-year-old woman with mild SLE disease activity provided first insights into both the dynamic characteristics of 12 h-to-12 h sTNF-R55 changes and the complex associations between sTNF-R55 and SLE symptoms under real-life conditions. By applying a combination of univariate ARIMA modeling and bivariate cross-correlational analyses on 112 12-h measurements, we were able to not only analyze the concurrent correlation but also the time-lagged effects and various cause-effect relations between the variables under study.

Univariate analysis revealed a second-order autoregressive process (AR [2]) in the urinary sTNF-R55 time series, indicating a circadian rhythm. Specifically, sTNF-R55 readings were higher during the day and lower during the night (Fig. 1a). This is in line with the idea that sTNF-R55 may be antagonistic to TNFα as production of pro-inflammatory cytokines such as IL-2, IL-6, IL-12, TNFα, and IFN-γ was found to be maximal during sleep, while the anti-inflammatory cytokine IL-10 reached its maximum during the daytime [19]. In addition to the circadian rhythm, univariate ARIMA modeling of the urinary sTNF-R55 time series revealed a stochastic seasonal first-order moving average process (SMA [1]) with a span of seasonality of 14 (s = 14), suggesting a weekly or circaseptan rhythm in the urinary sTNF-R55 time series as well (Fig. 1a). In a another integrative single-case study on a healthy woman [20], we had already found a circaseptan component in the urinary neopterin/creatinine ratio, an indicator of cellular immune activation [15]. Circaseptan rhythms in human biological data have been most extensively mapped in steroid metabolite excretion and are thought to be due to both anthropogenic (the social week) as well as natural geomagnetic influences [21, 22].

While the mean concentration of urinary sTNF-R55 over the entire observation period of this study (277 ± 183 pg/ml, see Table 1) proved to be normal [23], bivariate time series analyses revealed that the clear 12-h-to-12-h variations in urinary sTNF-R55 concentrations (range 52–861 pg/ml) were significantly related to the temporal changes of several SLE symptoms. This supports the notion that TNF-α and its soluble receptors are causally involved in SLE pathogenesis [5]. Regarding the dynamic interrelationship between urinary sTNF-R55 concentrations and urinary protein levels (no pathological proteinuria [<150 mg/d] [24], range from 0.07 to 6.38 mg/h, see Table 1), we found that increased urinary sTNF-R55 concentrations preceded decreased urinary protein levels by 36–48 h and that increased urinary protein levels preceded increased urinary sTNF-R55 concentrations by 24–36 h. Furthermore, increased urinary sTNF-R55 levels preceded increased oral ulcers by 36–48 h, and increased oral ulceration preceded decreased urinary sTNF-R55 levels by 36–48 h. In addition, increased urinary sTNF-R55 levels preceded decreased facial rash by 36–48 h and followed increased body temperature after 36–48 h.

Because of the equivocal evidence in the literature regarding the role of TNFα and its soluble receptor subtypes in SLE [2–7], we refrain from discussing each of these findings in detail and, instead, try to understand the complex relationships between urinary sTNF-R55 concentrations and SLE symptoms by taking a broader view of the cross-correlational findings summarized above.

The long time intervals between changes in urinary sTNF-R55 concentrations and changes in urinary protein levels, oral ulceration, facial rash, and body temperature of up to 48 h are similar to those shown in other publications by our working group that apply the same kind of research design as in the current study [8, 11, 12]. Such long time intervals may be due to long-term neuroimmunological regulation circuits in which processes are coupled and operate over various time scales [25].

Moreover, by showing significant cross-correlation coefficients in both directions of effect (positive and negative lags), strong negative feedback loops between sTNF-R55 activity and SLE symptoms can be posited, in which a positive (negative) anomaly in one process at some earlier time tends to become negative (positive) after having interacted with the other process, and vice versa [26]. This applies especially to the relation between urinary sTNF-R55 and urinary protein (see Fig. 2a), and between urinary sTNF-R55 and oral ulceration (see Fig. 2b). But even in Fig. 2c, several non-significant positive correlations in the direction from facial rash to sTNF-R55 (negative lag) are recognizable. This indicates the possible existence of a negative feedback loop between sTNF-R55 and facial rash, and suggests that, to evaluate the findings of our study, we need to consider both significant coefficients and temporal patterns of (non-significant) coefficients [8]. The finding of an increase in oral ulceration and a decrease in facial rash following increases in sTNF-R55 concentrations might indicate different pathomechanisms of mucosal and cutaneous manifestations in this SLE patient.

The following is an attempt to read the cross-correlograms seen in Fig. 2a, b as negative feedback mechanisms. For on, elevated sTNF-R55 levels might have initially inhibited protein clearance from circulation after 36–48 h (positive lag and negative correlation in Fig. 2a), and decreased protein clearance could have then resulted, either per se or via an as yet unknown counterregulatory mechanism, in decreased sTNF-R55 concentrations 24–36 h later (negative lag and positive correlation in Fig. 2a). This corresponds well with results from animal studies showing that the induction of renal impairment results in decreased renal clearance of TNF-α and increases in circulating TNF–TNF-R complexes [27]. Moreover, in the patient of this study, elevated sTNF-R55 levels might have first triggered oral ulcerations after 36–48 h (positive lag and positive correlation in Fig. 2b), and oral ulcerations, in a counterregulatory mode, could have then activated agents to lower sTNF-R55 levels 24–36 h later (negative lag and negative correlation in Fig. 2b). In future studies, the use of multivariate time series statistics (vector autoregressive modeling, impulse response analysis) will allow us to properly disentangle the temporal sequence of the events within feedback processes [28].

The current investigation showed that increases in body temperature preceded increases in urinary sTNF-R55 concentrations. However, in the opposite direction of effect, it neither showed that urinary sTNF-R55 levels preceded changes in body temperature nor that urinary sTNF-R55 concentrations were significantly related to fatigue, weakness, and small joint pain in any direction of effect (see Table 2). This lack of association in our study may be attributable to the VAS scale used to determine SLE symptoms; it is a simple measure that could be supplemented by more differentiated psychological questionnaires. A further limitation of this study is that no objective measurement of the specific SLE symptoms was applied (e.g. number of mucosal and skin lesions per 12 h). Moreover, other inflammatory parameters besides sTNF-R55, such as interleukin-6 (IL-6), IL-1α or IL-1β, which have been shown to be associated with SLE symptoms and sickness behavior [29], could be used in future studies. In previous publications dealing with this single-case study, we have already shown that emotionally positive and negative daily incidents led to cyclic concentration changes in urinary neopterin with temporal delays of up to 144 h [11, 12]. Moreover, consumption of alcoholic beverages differentially interfered with the patient’s urinary neopterin levels [13]. Regarding the limitations mentioned above, it should be noted that the current study is exploratory in nature, and further studies on patients with stable as well as full-blown SLE disease activity will be needed before firm conclusions can be drawn.

## Conclusions

A considerable advantage of the study design is the opportunity it offers to account for the real-time dynamic characteristics of cytokines and the nature of cytokine-SLE symptom interdependencies, thereby providing basic insights into the dysfunctional physiology of SLE. This study showed for the first time that sTNF-R55 dynamics in SLE are characterized by both circadian and circaseptan rhythms. Moreover, it demonstrated that effects between sTNF-R55 and SLE symptoms can be delayed by up to 48 h and that real-life long-term feedbacks between sTNF-R55 and SLE symptoms may exist. As a consequence, this study calls the use of pre-post designs in clinical research into question by demonstrating that (1) a lack of correlation between cytokine level and SLE symptoms in pre-post designs does not automatically mean that the cytokine is not connected with disease activity; (2) a single significant correlation between cytokine level and SLE symptom in pre-post designs does not allow inferences on the functional role of a cytokine; and (3) neglecting the dynamic characteristics of the relation between cytokine level and SLE symptom (e.g. differently lagged effects in different patients) may be connected with inconsistent findings in pre-post designs.

## Footnotes

1 We dedicate this work to the late Franz Halberg, a pioneer in the area of chronobiology.
