# Erratum to: Cause-effect relations between 55 kD soluble TNF receptor concentrations and specific and unspecific symptoms in a patient with mild SLE disease activity: an exploratory time series analysis study

**DOI:** 10.1186/s13104-017-2419-x

**Published:** 2017-02-17

**Authors:** Christian Schubert, Julia Haberkorn, Francisco M. Ocaña-Peinado, Paul König, Norbert Sepp, Mirjam Schnapka-Köpf, Dietmar Fuchs

**Affiliations:** 1 Clinical Department of Medical Psychology, Innsbruck Medical University, Schöpfstraße 23a, 6020 Innsbruck, Austria; 2 Department of Statistics and Operations Research, University of Granada, Granada, Spain; 3 Clinical Department of Internal Medicine, Innsbruck Medical University, Innsbruck, Austria; 4 Clinical Department of Dermatology, Innsbruck Medical University, Innsbruck, Austria; 5 Central Institute of Medical and Chemical Laboratory Diagnostics, University Clinics, Innsbruck, Austria; 6 Division of Biological Chemistry, Biocenter, Innsbruck Medical University, Innsbruck, Austria

## Erratum to: BMC Res Notes (2015) 8:465 DOI 10.1186/s13104-015-1398-z

After publication of the original article [1], it came to the authors’ attention that there was an error in the Results section of the abstract. ‘Circasemiseptan’ was incorrectly used in the first sentence; the correct word is ‘circaseptan’.
